# Cultivated Land Demand and Pressure in Southeast Asia from 1961 to 2019: A Comprehensive Study on Food Consumption

**DOI:** 10.3390/foods12193531

**Published:** 2023-09-22

**Authors:** Yuting Qin, Jiayue Tang, Tanglu Li, Xin Qi, Dan Zhang, Sijia Wang, Fei Lun

**Affiliations:** 1 College of Land Science and Technology, China Agricultural University, Beijing 100193, China; yuting.qin@cau.edu.cn (Y.Q.); tangjiayue@cau.edu.cn (J.T.); litanglu0206@cau.edu.cn (T.L.); 2020317010317@cau.edu.cn (X.Q.); wangsijia0821@126.com (S.W.); lunfei@cau.edu.cn (F.L.); 2 Institute of Geographic Sciences and Natural Resources Research, Chinese Academy of Sciences, Beijing 100101, China

**Keywords:** food consumption, cultivated land footprint, cultivated land pressure, Southeast Asia

## Abstract

Southeast Asia plays a crucial role in global food production and trade, yet it grapples with challenges related to food security, regional stability, and security. Cultivated land is the material foundation for ensuring food production. With the development of society and the economy, people’s food consumption has undergone significant changes. This paper employs a comprehensive approach to analyze trends in food consumption, the cultivated land footprint, and associated land pressures in Southeast Asia over the period 1961–2019. The main findings are as follows: (1) Between 1961 and 2019, the total food consumption in Southeast Asia surged by 3.1 times. Notably, the proportion of livestock-based foods increased steadily from 6.62% in 1961 to 16.82% in 2019. (2) Due to advancements in agricultural productivity across Southeast Asia, the cultivated land footprint for food consumption only increased by 0.7 times, showcasing a diminishing demand for grain-cultivated land. (3) On the whole, the pressure of food consumption on cultivated land in Southeast Asia is on the decline, albeit with considerable variations among different countries. The Philippines is facing a relative undersupply, whereas Thailand has experienced the lowest cultivated land pressure. (4) Encouraging a shift towards a Mediterranean-style diet, aligned with existing dietary patterns, holds promise for reducing future pressures on cultivated land and promoting better health outcomes for the populace in Southeast Asia.

## 1. Introduction

Cultivated land forms the bedrock for ensuring global food production. As of 2020, the cultivated land area worldwide accounted for 12% of the total, effectively meeting the food needs of 7 billion people [1,2,3]. However, with societal and economic development, significant shifts have occurred in people’s food consumption patterns, particularly with the increasing consumption of livestock-based foods [4]. If current dietary trends persist, it is projected that by 2050, approximately 40% of the global population will face challenges in securing sufficient land and water to meet their food requirements [5]. Consequently, as the conflict between human needs and available land intensifies, achieving harmonious development between people and the land has become a prominent concern within the academic community [6,7].

In response to the escalating global population and evolving food dietary requirements, researchers have delved into land demand assessments based on food consumption [8]. Gerbens-Leenes [9,10] introduced the concept of virtual land, positing that the aggregate land necessary for a specific type of food is determined by both its specific land demands and the quantity consumed. This notion sheds light on the substantial impact of food consumption patterns on the corresponding land needs, leading to significant regional and intergenerational disparities in Western countries. For instance, in the ecologically fragile region of Tibet, Zhang et al. quantitatively assessed the spatial and temporal patterns of Tibet’s Land Carrying Capacity (LCC) during the period 2000–2019, which revealed localized overload and increasing tension in the load on land (LoL) resources in Tibet [11]. In addition, other researchers have explored food consumption-driven land demands at varying scales, encompassing global, national, and local levels [12,13,14,15]. Furthermore, utilizing the Multi-Regional Input-Output (MRIO) models, researchers have evaluated the land demands for consumption in Australia, Belt and Road countries, and China [16,17,18], respectively. The diversified evolution of food dietary requirements [19] has contributed to a steady rise in the demand for cultivated land to meet the dietary needs of residents [20]. However, global cultivated land area is dwindling, exerting more pressure on land requirements for food consumption. Simultaneously, the varying levels of socioeconomic development and natural resource endowment have exacerbated the spatial mismatch between global food production and land resources, impacting the sustainable utilization of cultivated land resources [21]. Hence, scrutinizing land demands and pressures amid the evolution of residents’ food consumption patterns holds immense significance and value in achieving the sustainable development of cultivated land [22,23,24].

Despite the favorable natural conditions for agricultural production in Southeast Asia, inadequate agricultural infrastructure, outdated production technology, and recurrent natural disasters have kept local agricultural productivity significantly below the global average [25,26,27,28]. The population in Southeast Asia is substantial, reaching 656 million and accounting for approximately 9% of the global population. Consequently, food consumption among residents has been consistently rising. However, the instability in food production and supply has left approximately 126 million people in Southeast Asia moderately or severely food insecure, while another 48.8 million people face the threat of hunger, constituting approximately 6.4% of the global starving population [27,28]. These challenges have significantly impacted the health of the local populace, including a high prevalence of stunting for 27.4% of the children under 5 years of age. Moreover, food security challenges in the region have triggered issues such as human trafficking [29], illegal migration [30], and piracy [31], posing serious threats to local and even global development and stability [32,33]. The recent shifts in food consumption patterns in Southeast Asia have led to an increased demand for land resources. Consequently, food security and the pressure on cultivated land in Southeast Asia have become critical global concerns [34,35]. For instance, Kastner et al. investigated the total land requirement for food production in the Philippines from 1910 to 2003 [36]. However, current studies on food consumption and land demand in Southeast Asia have primarily focused on the national scale, with comparative research across countries in the region and exploration of the impacts of changing food consumption patterns on local cultivated land pressures being relatively scarce.

Thus, employing the concept of the cultivated footprint of food consumption, which refers to the area of cultivated land required to produce a unit of food, which is equal to the amount of cultivated land for food consumed divided by the amount of food consumed, this study aimed to: (1) examine food consumption and its structural changes in each country in Southeast Asia from 1961–2019; (2) analyze the cultivated footprints of different foods and explore the cultivated land demands in each country in Southeast Asia; (3) analyze the changes in cultivated land pressures due to food consumption in each country from 1961–2019; and (4) explore future food consumption and its cultivated land demand in Southeast Asia.

## 2. Methodology

### 2.1. Study Area

Southeast Asia is located in the southeast part of Asia and includes 11 countries (Vietnam, Laos, Cambodia, Thailand, Burma, Malaysia, Singapore, Indonesia, Brunei, The Philippines and Timor-Leste). All of these countries are participants and advocates of the Belt and Road Initiative [37]. With 140.113 million hectares of total agricultural land, agriculture is an important economic pillar in the Southeast Asian region. In 2021, agriculture produced approximately 10% of the GDPs (gross domestic product) of Cambodia, Indonesia, Laos, The Philippines, Timor-Leste, and Vietnam, which is far higher than the world average (4.3%). Southeast Asia is an important exporter of cereals in the world, with Thailand and Vietnam accounting for 22% and 12% of global rice exports, respectively [25,38]. Southeast Asia has a large and dense population. In 2021, its total population reached 675 million. According to the latest FAO data, the total food consumption in Southeast Asia in 2019 was 1.315 billion tons, and the per capita food consumption was 275.09 kg/person/year. In our study, we only focused on nine Southeast Asian countries—Cambodia, Indonesia, Laos, Malaysia, Myanmar, The Philippines, Thailand, Timor-Leste, and Vietnam—due to data availability.

### 2.2. Methods

Data on the food consumption levels [39,40], yields [41], cultivated land areas [1], and populations [41] in Southeast Asia from 1961 to 2019 were sourced from FAOSTAT. This study examined 14 categories of food as research subjects for both Southeast Asia as a whole and the individual countries within the region. These categories included wheat, maize, rice, soybeans, starchy products and roots, vegetables, other plant-based foods, beef, mutton, pork, poultry, eggs, dairy products, fish, and shellfish. The category “other plant foods” encompassed barley and its products, rye and its products, oats, millet and its products, other cereals, sugar crops, pulses, tree nuts, oil crops, stimulants, and spices. The specific data on caloric content per unit weight of various foods were sourced from official USDA data [42], detailed in Appendix A (Table A1).

The proportions of meat, eggs, and milk in the category of animal-based food were derived from studies conducted by Pradeep [43], APA [44], Clark [45], Craig Thomas [46], and other individuals or organizations, as outlined in Appendix A (Table A2). The predictions regarding future population data were sourced from the United Nations Population Division [47,48]. Additionally, the future food consumption structure was primarily based on the 2019 FAO statistics, the average recommended intake of various foods in Southeast Asian dietary guidelines [49], and the Mediterranean Diet Pyramid [50]. The formula for the future food consumption structure is the same as that for present food consumption structure. The calculation method employed was as follows:

#### 2.2.1. Cultivated Land Demands for Food Consumption

This study used food consumption data, crop yield data, and livestock food meat (or eggs and milk)/feed ratio data from the FAO to calculate and analyze the cultivated land demands and footprints for food consumption in Southeast Asian countries from 1961 to 2019 to further analyze the effects on productivity levels and man–land relationships in Southeast Asia. The specific parameters included demands for cultivated land that were consumed by different kinds of food, total land demands, per capita land demands, and cultivated land footprints for food consumption. The calculation formulas for the main parameters are as discussed below.

The cultivated land demand for food consumption refers to the area of cultivated land needed to meet residents’ food demands, and this is typically calculated by dividing the amount of food consumed by the yield of food produced per unit of cultivated land. Given the unique nature of livestock products, the cultivated land demand for food consumption is usually converted to the area of cultivated land required to produce the grain necessary for the production of a certain livestock product. This conversion is achieved through the introduction of the “meat (or egg/milk)/feed ratio” coefficient. The livestock product consumption amounts are multiplied by this coefficient to ascertain the amount of food necessary to produce a specific quality of livestock product. This value is then divided by the unit yield of grain to determine the area of cultivated land needed for livestock product consumption. The concept of the cultivated land footprint for food consumption, proposed by Gerbens-Leenes, refers to the area of cultivated land required to produce food per unit of quality. It is calculated by dividing the amount of cultivated land for food consumed by the amount of food consumed. In this study, the cultivated land footprint for food consumption was introduced to capture shifts in agricultural productivity and variations in the amount of cultivated land required for different food consumption patterns in Southeast Asia.

This study utilized food consumption data, crop yield data, and livestock food meat (or eggs and milk)/feed ratio data from the FAO to calculate and analyze the cultivated land demands and footprints for food consumption in Southeast Asian countries from 1961 to 2019. The objective was to further analyze the impacts on productivity and the human–land relationship in Southeast Asia. The specific parameters considered included the demands for cultivated land consumed by different types of food, total land demand, per capita land demand, and the cultivated land footprint for food consumption. The main calculation formulas for these parameters are as follows:

(1) $$PD_{i}=\frac{TC_{i}}{Y_{i}},$$

(2) $$AD_{i}=\frac{TC_{i}}{Y_{c}}\times \alpha_{i},$$

(3) $$Y_{c}=\frac{Y_{wheat}+Y_{maize}+Y_{rice}}{3},$$

(4) $$Footprint_{i}=\frac{D_{i}}{TC_{i}},\;\mathrm{and}$$

(5) $$D\_capita_{i}=\frac{D_{i}}{P},$$

where $PD_{i\;}$ represents the cultivated land demand for the crop food *i*, $Y_{i\;}$ represents the yield per unit of land for the crop food *i*, $AD_{i\;}$ represents the cultivated land demand for the livestock food *i*, $Y_{c\;}$ represents the average yield for grains (i.e., wheat, maize, and paddy rice), $\alpha_{i}$ represents the ratio of meat (or eggs and milk) required to feed the livestock food *i*, $Footprint_{i}$ represents the cultivated land footprint for the food *i*, $D_{i\;}$ represents the cultivated land demand for the food *i*, and $D\_capita_{i}$ represents the per capita cultivated land demand for the food *i*.

#### 2.2.2. Cropland Pressure for Local Food Consumption

The cultivated land pressure for food consumption is defined as the ratio of cultivated land demand attributed to food consumption to the actual cultivated land area. By incorporating FAO’s cultivated land area data, this study calculated the land pressure in Southeast Asian countries from 1961 to 2019 and analyzed the human-land contradiction scenario. The formula for computing the land pressure is as follows:

(6) $$P=\frac{\sum D_{i}}{\mathrm{Area}\_\mathrm{Cropland}},$$

where P represents the pressure value of food consumption on the current land resources and Area_Cropland denotes the existing cultivated land area. If the resulting ratio is greater than 1, it signifies that the present cultivated land resources in the region can adequately provide for its residents in terms of food consumption, indicating a relatively high level of food self-sufficiency. Conversely, if the ratio is less than 1, it signifies that the existing cultivated land resources fall short of meeting the current dietary structure, highlighting a relatively high degree of dependence on external sources.

## 3. Results

### 3.1. Food Consumption in Southeast Asia for the Period 1961–2019

**Figure 1:**
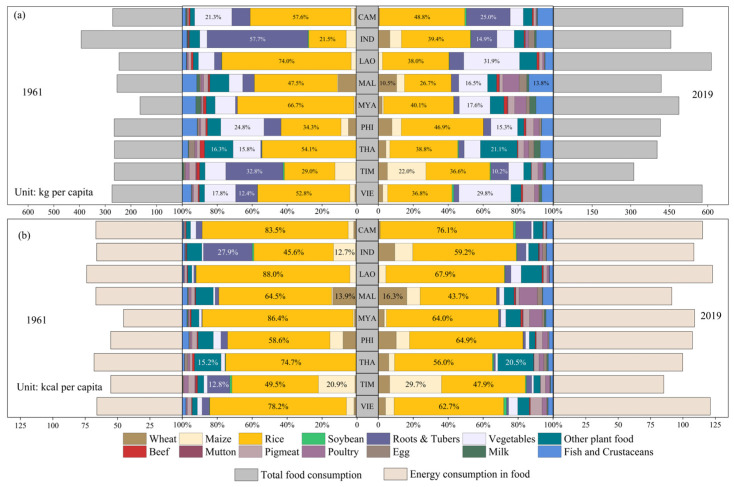
Food consumption, food calories, and their compositions in Southeast Asia during the period 1961–2019. (**a**) Food consumption and its composition. (**b**) Food calories consumption and its composition.

As the local economies in Southeast Asia have developed, there has been a noticeable increase in food consumption per capita. In 2019, per capita food consumption reached 465.18 kg, providing approximately 1.09 × 10^6^ kcal per person (Figure 1). Moreover, the region has experienced a shift towards greater food diversity in recent years. While plant-based foods continue to play a predominant role in local food consumption, the consumption of livestock-based foods has been steadily rising. Notably, poultry consumption in 2019 was approximately eight times higher than that in 1961. This increase in consumption led to livestock foods accounting for 11% of food calories in 2019, nearly double the percentage observed in 1961. However, it is important to note that Southeast Asia’s share of food calories from livestock foods continues to lag behind the global average of 17.6%. Local cereal consumption has also seen a significant increase, with a per capita increase of 50.29 kg over the past 50 years. Rice, in particular, contributes the most to food calories, making up 60.72% of the total. Although vegetable consumption is on the rise in Southeast Asia, its contribution to food calories remains relatively low, at just 2%, due to its lower calorie content. Given the coastal nature of many countries in the region, aquatic product consumption holds a relatively higher share of local food consumption in Southeast Asia.

Food dietary requirements and their compositions vary among Southeast Asian countries, largely due to the different economic levels. Laos boasts the highest food consumption per capita at 613.85 kg, providing 1.23 × 10^6^ kcal, although its consumption of livestock-based foods is relatively low, constituting only 6% of total food calories. Conversely, Timor-Leste had the lowest food consumption per capita in 2019, at 307.11 kg, with a calorie intake of 0.85 × 10^6^ kcal, amounting to 78% of the regional average. Furthermore, its food composition has remained relatively stable over the past 60 years. The most substantial increase in per capita food consumption occurred between 1961 and 2019, particularly in the consumption of livestock-based foods. To provide a more detailed perspective, livestock food consumption increased nearly five-fold, reaching 135.01 kg per capita in 2016, with poultry consumption alone amounting to 32.29 kg. Food consumption has become increasingly diverse among Southeast Asian countries, with Malaysia and Myanmar standing out. These two countries exhibit relatively higher livestock food consumption compared to others, with per capita consumption levels of 136.18 kg and 140.86 kg, respectively, in 2019, contributing to 23% and 18% of the total food calories, respectively.

### 3.2. Land Footprints of the Different Foods in Southeast Asia

**Figure 2:**
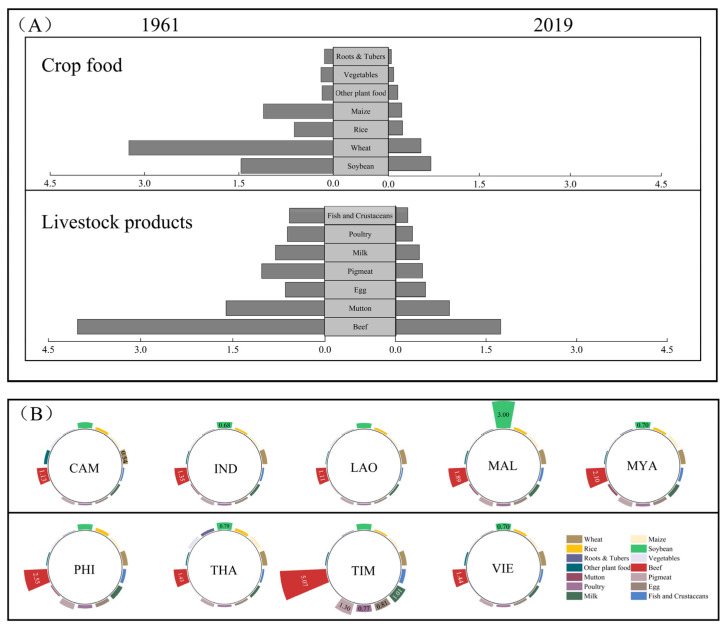
The cultivated land demand for food consumption in southeast Asia for the different food types in 1961 and 2019 (**A**) and for the different countries (**B**).

During the period 1961–2019, advancements in technology and institutional innovations led to continuous improvements in agricultural efficiency across Southeast Asia. Consequently, the average land demand for food consumption decreased by 56%, from 1.16 ha/t to 0.51 ha/t. However, substantial disparities exist in the land footprints among various food types (Figure 2). Livestock products generally have a land footprint that is 1.43 times larger than that of crops. Within livestock products, white meat (including fish, shrimp, crab, and poultry) is relatively highly consumed in Southeast Asia. However, in comparison to red meat (pork, beef, and mutton), it has a lower land footprint, with values of 0.20 ha/t and 0.22 ha/t, respectively. Notably, beef has the highest land footprint at 1.44 ha/t. Compared to 1961, the average land footprint for livestock products was reduced significantly by 1.33 ha/t, with an approximate decrease of 70%. Among these, the reduction in the land footprint due to mutton consumption was the highest, at 95%.

In 2019, the land footprint for crops ranged from 0.09 to 0.70 ha/t, with vegetables having the smallest footprint and soybeans the largest [51]. Over the period 1961 to 2019, the land footprint for cereals, i.e., wheat, maize, and rice, the primary sources of calories in Southeast Asian diets, saw significant declines of 83%, 80%, and 62%, respectively, although they remained higher than the world average (0.29 ha/t, 0.17 ha/t, and 0.22 ha/t, respectively). Differences in the land footprints for food consumption exist among the countries of Southeast Asia. Timor-Leste, being one of the world’s poorest countries, exhibits much higher land footprints for almost all foods compared to the regional averages. In particular, the land footprint for beef in Timor-Leste is notably high, reaching 5.07 ha/t. Moreover, Malaysia has a relatively high soybean land footprint of 3.00 ha/t, surpassing those of other countries and the Southeast Asian average.

### 3.3. Cultivated Land Demands for Food Consumption in Southeast Asia

With the increases in population and per capita food consumption in Southeast Asia, its total food consumption increased four-fold during the period 1961–2019, and its total calorie intake from food increased by 78%, from 6.26 × 10^5^-kcal to 1.09 × 10^6^ kcal. The demand for cultivated land for food consumption grew from 3.20 × 10^7^ ha to 6.68 × 10^7^ ha, with a 2.09-fold increase. Thanks to the increased crop yields, the per capita cultivated land demand for food consumption has dropped by 43%. In 2019, a significant portion of the agricultural demand for food consumption in Southeast Asia was attributed to rice (42%) and a smaller fraction to livestock products (26%). However, compared to plant-based foods, the demand for cultivated land for the food consumption of livestock products notably increased, particularly the demand for poultry, which surged by 8.59 times.

**Figure 3:**
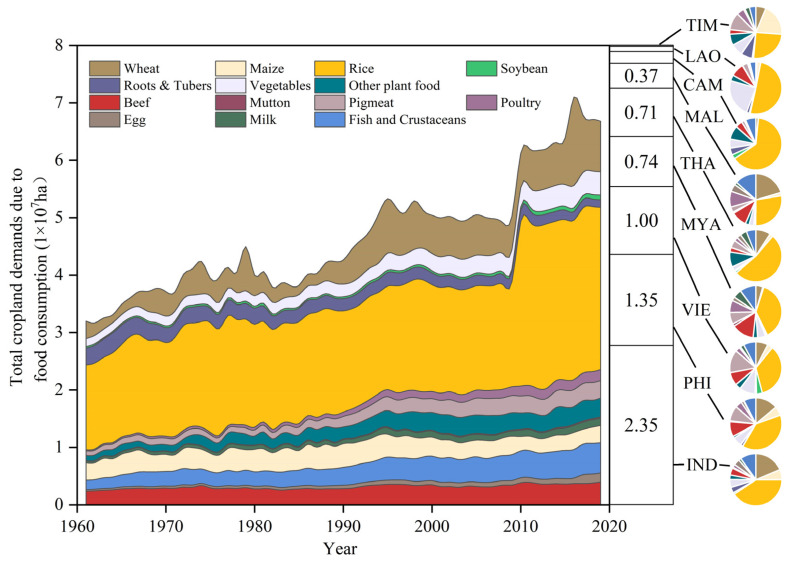
Cultivated land demands for different food consumption levels in Southeast Asia from 1961 to 2019 (left), total cultivated land demand for food consumption in 2019 (middle), and proportions of the cultivated land demand for the various types of food in each country in 2019 (right).

**Figure 4:**
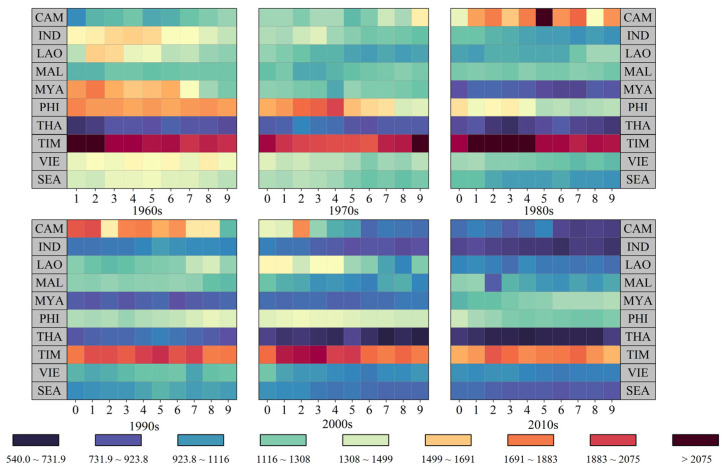
Per capita cultivated land demands for food consumption in Southeast Asia and each country during the period 1961–2019 (m²/person).

Although Timor-Leste has the highest cultivated land footprint for food consumption among Southeast Asian countries, its smaller population and lower per capita food consumption have resulted in a cultivated land demand of only 1.80 × 10^5^ ha. Conversely, Indonesia, with its similar cultivated land footprint, exhibited the highest total food consumption among all the countries, making it the country with the highest cultivated land demand for food consumption. Beef holds the highest cultivated land footprint in all of the countries except Malaysia (Figure 3). Beef consumption significantly influences the structure of cultivated land demand for food consumption. Therefore, Myanmar, with the highest per capita beef consumption ratio (approximately 2%), sees its cultivated land demand for food consumption accounting for 14%, whereas Cambodia’s cultivated land demand for all livestock product consumption accounts for only 13%. Other countries with lower beef consumption exhibit a lower proportion of beef in the cultivated land demand for food consumption (Figure 4). Malaysia and Myanmar, with the highest per capita consumption levels of livestock products, also have the highest proportions of cultivated land demands for livestock product consumption among all countries, at 43% and 48%, respectively.

Per capita cultivated land demand for food consumption refers to the amount of land needed by a country to meet the food consumption needs of its residents. Amidst a general increase in population, calculating per capita cultivated land demand for food consumption and studying its changing trends can better illustrate the conflict between population growth and limited land resources, guiding future population and agricultural policy adjustments. The per capita cultivated land demand in each of the studied countries exhibited distinct changing characteristics. During the study period, the per capita cultivated land demands for food consumption decreased in all countries, with Indonesia experiencing the largest decrease (56%). Conversely, Thailand saw an increase from 614.45 m^2^/person in 1961 to 651.29 m^2^/person in 2019. Despite this rise, Thailand had the smallest per capita cultivated land demand for food consumption in 2019 when compared to other Southeast Asian countries. On the other hand, in 2019, Timor-Leste had the highest per capita cultivated land demand for food consumption, at 1549.98 m^2^/person. Between 1979 and 1998, Cambodia experienced relatively high and fluctuating per capita demands for cultivated land, with a maximum fluctuation range of nearly 900 m^2^/person during this period. From 2000 to 2019, Myanmar’s per capita demand for cultivated land increased by 453.09 m^2^/person, while the rest of Southeast Asia witnessed declines.

### 3.4. The Trends in Cultivated Land Pressure for Food Consumption in Southeast Asia

**Figure 5:**
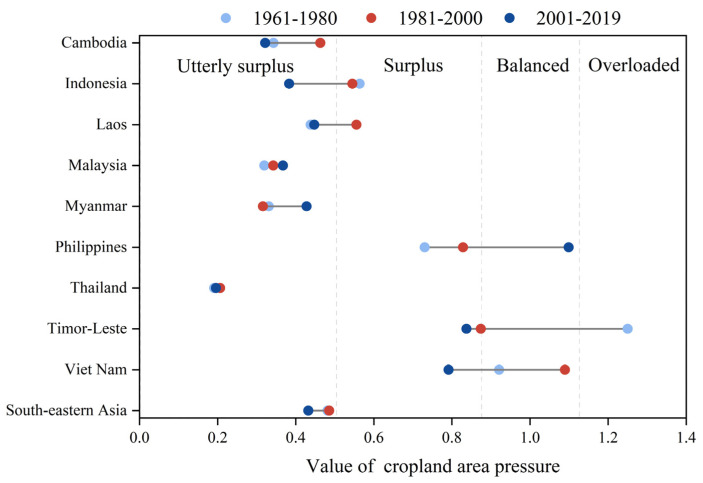
Changes in the cultivated land pressure for food consumption in Southeast Asia and in each country.

The cultivated land pressure in Southeast Asia ranged between 0.4 and 0.5 during the period 1961–2019 (Figure 5), suggesting that grain production in the region could adequately meet the food consumption demands. Notably, the cultivated land demand pressures in Cambodia, Indonesia, Laos, Malaysia, Myanmar, and Thailand remained below 0.6. Among these countries, Thailand, a significant global food producer, consistently maintained the lowest cultivated land pressure for food consumption, hovering at approximately 0.2. Conversely, the Philippines, Timor-Leste, and Vietnam experienced relatively high levels of cultivated land pressure for food consumption. Additionally, each country exhibited distinct changing patterns influenced by economic development, agricultural production efficiency, and population dynamics. Specifically, between 1961 and 2019, the cultivated land demand pressure for food consumption in The Philippines steadily increased from 0.73 (1961–1980) to 0.83 (1981–2000). From 2001 to 2019, the cultivated land pressure for food consumption in The Philippines exceeded 1.0, reaching just under 1.10. This indicated that during this period, The Philippines’ domestic food production could no longer meet its residents’ food consumption needs, necessitating increased food imports to fulfill the population’s requirements. In contrast to The Philippines, East Timor witnessed a continuous decline in cultivated land pressure for food consumption. From 1961 to 1980, the cultivated land pressure for food consumption in East Timor could not meet domestic consumption, measuring only 0.84 during the period 2000 to 2019. Moreover, Vietnam experienced a cultivated land pressure caused by food consumption exceeding 1.0 during the period 1981–2000. However, advancements in agricultural production technology have eased the cultivated land pressure for food consumption, resulting in a measurement of only 0.79 during the period 2001–2019.

## 4. Discussion

### 4.1. Economic Development, Food Consumption and Cropland Demand

With the advancements in the social economy, variations in food consumption and dietary structures among residents in Southeast Asia have emerged. Notably, from 2000 to 2019, the consumption of livestock products by residents increased in Southeast Asian countries, aligning with previous research findings [52,53]. A significant contributing factor has been the progress in agricultural technology, leading to substantial reductions in the production costs and prices of livestock products [54]. Moreover, rising incomes have influenced residents’ dietary preferences, prioritizing health considerations and a diverse range of food choices [55]. The proliferation of the internet has further facilitated residents in accessing food with higher nutritional value or specialized regional cuisines [56]. However, due to varying consumption habits and economic growth rates, the rise in livestock product consumption has differed across the studied countries.

Myanmar, with its swift economic growth, has exhibited a notable increase in livestock product consumption. Over the past two decades, its consumption of livestock product calories has surged by 4.9 times, while Thailand observed a relatively modest increase. Furthermore, there were significant variations in the increases in livestock product consumption among the different countries, with seafood consumption registering prominent rises in most nations. Despite the concurrent increases in livestock product consumption and population, enhanced agricultural production efficiency has mitigated the cultivated land footprint. Consequently, the local land demand for livestock product consumption did not witness a significant increase, and in some cases, it even decreased. For instance, Myanmar experienced a decrease of approximately one-quarter in its livestock product farmland footprint between 2000 and 2019, despite a 4.9-fold increase in livestock product consumption and a 1.16-fold increase in population. This resulted in an approximate 4.12-fold increase in its per capita demand for cultivated land. Notably, advancements in productivity and shifts in consumption patterns have led to decreases in the total cultivated land demand for food consumption in Cambodia, Laos, and Thailand from 2000 to 2019. Therefore, promoting the development of agricultural technology and enhancing agricultural production efficiency can effectively mitigate the land demand for residents’ food consumption, contributing to the sustainable utilization of local land.

### 4.2. Future Food Consumption, Cultivated Demand, and Local Population

Food dietary requirements and population are two primary factors influencing the demand for cultivated land for food consumption [9,13,57]. As the Southeast Asian social economy continues to develop, future changes in population and food consumption patterns will significantly impact the cultivated land pressure for food consumption in the region. In light of this, this study combined FAO population forecast data with different food consumption scenarios to explore the future land demand situation in Southeast Asia. The scenarios are defined as follows: scenario 1 represents the current situation, scenario 2 reflects the recommended dietary guidelines, and scenario 3 follows the Mediterranean diet (Appendix A, Table A3 and Table A4).

Despite the recent increase in livestock product consumption in Southeast Asia, staple foods remain the primary sources of calories. The per capita consumption of staple foods has significantly exceeded that of the recommended guidelines and the Mediterranean diet, while livestock product consumption, especially eggs and milk, has fallen below the levels recommended by these dietary guidelines. Anticipating continued socio-economic development, the population of Southeast Asia has been projected to increase by 19% in 2050 and 11% by 2100 compared to the population in 2020. Thus, within scenario 1, the cultivated land pressures in various countries will rise in line with population growth from 2020 to 2100. Timor-Leste, in particular, will experience the most significant increase in cultivated land pressure, with an approximate 80% increase, causing a shift from land oversupply to short supply. Conversely, Thailand will observe a decrease in both population and cultivated land demand for food consumption by 1.55 × 10^6^ ha in 2020, resulting in a corresponding decrease in cultivated land pressure. In scenario 2, the total demand for cultivated land for food consumption in Southeast Asia will rise by 1.36 × 10^7^ ha, resulting in a pressure increase of 0.09. Notably, the cultivated land demand for food consumption and the corresponding pressure will increase across most countries, except for Thailand and Myanmar.

Distinct from the other scenarios, the Mediterranean diet features a lower consumption of foods with a high cultivated land footprint, particularly for livestock products, and a higher consumption of foods with a low cultivated land footprint, including vegetables. Consequently, the demand for cultivated land for food consumption under the Mediterranean diet is relatively low. Compared to the situation in 2020, the cultivated land demand for food consumption in Southeast Asia would decrease by 6.48 × 10^6^ ha under the Mediterranean diet scenario, leading to a reduction in the cultivated land pressure of 0.06. Simultaneously, many countries would experience declines in their cultivated land demands for food consumption, to varying degrees. Therefore, as overall cultivated land pressure is expected to increase in the future, adjusting diet structure in a rational manner can effectively alleviate this pressure, promoting both the well-being of local residents and sustainable land usage.

### 4.3. Limitation and Future Studies

While we have extensively discussed shifts in food consumption and land demands across various countries in Southeast Asia, it is important to acknowledge certain limitations in this study due to the availability of cultivated land data. Southeast Asia holds a significant position as a global fruit production hub; however, challenges in obtaining precise orchard area data have led to the absence of an analysis on land pressure related to fruit consumption in the region. Moreover, although cultivated land in Southeast Asia generally caters to the consumption needs of domestic residential areas, the evolving landscape of economic globalization has prompted concerns regarding the sustainable use of agricultural land. Southeast Asia’s pivotal role as a major producer and exporter of agricultural products accentuates the need to explore how to effectively preserve the sustainable use of agricultural land. Therefore, in future studies, integrating an analysis of import and export situations into the evaluation of cultivated land demands and pressures in Southeast Asia is imperative for a comprehensive and precise understanding of the land demand scenario.

## 5. Conclusions

As the social economy in Southeast Asia continues to develop, both per capita and total food consumption among residents are on the rise. The food consumption levels across various countries in the region are diversifying, and they are notably marked by increases in the proportions of calories from livestock product consumption. Additionally, owing to varying social and economic levels among nations, the increases in the proportions of calories consumed from livestock products and the changes in food consumption have differed from 1961 to 2019.

Improvements in agricultural efficiency have significantly reduced the amount of cultivated land needed to produce a unit of food. For the period 1961 to 2019, with the advancements in agricultural technologies and systems in Southeast Asia, the average cultivated land footprint for food consumption in the region decreased by 56%. Cereal, being the main source of food calories for Southeast Asian residents, experienced the most substantial decline in cultivated land footprint. Consequently, total food consumption in Southeast Asia increased four-fold during this period, while the demand for cultivated land increased by only 1.09 times. Food consumption was the primary factor influencing cultivated land demand, except for beef and soybeans, where the food footprints varied among the countries. This led to Timor-Leste having the lowest cultivated land demand for food consumption in 2019 while Indonesia had the highest. Additionally, countries such as Malaysia and Myanmar, with their higher proportions of animal food consumption, had the highest demands for land in terms of livestock product consumption. The increases in agricultural production efficiency also effectively eased the conflict between population growth and land availability, resulting in declines in the per capita farmland demands across all countries during the study period.

Throughout the study period (from 1961 to 2019), the cultivated land demand for resident food consumption in Southeast Asia consistently remained lower than the actual cultivated land. The cultivated land pressure value exhibited a downward trend, except for the upward trends observed for Malaysia, Myanmar, and The Philippines. Between 2001 and 2019, except for The Philippines, which faced a relative insufficiency in cultivated land, other countries experienced oversupplies, with Thailand having the lowest demand. In the foreseeable future, alterations in consumption patterns will be the key variable affecting the overall land requirements for food. The increased consumption of foods associated with affluent lifestyles will drive greater demands for land. To alleviate the pressure on cultivated land resulting from food consumption and to steer nutritional transformation in Southeast Asia towards a healthier and environmentally friendly direction, appropriate structural adjustments based on the Mediterranean diet could be pursued. Changes in consumer behaviors at the household level can be powerful options for reducing the utilization of natural resources such as agricultural land.

## Data Availability

The data used to support the findings of this study can be made available by the corresponding author upon request.

## Appendix A

**Table A1:** Calories in different foods.

Food Type	Calorie (kcal/100) g
Wheat	339
Maize	368
Rice	357
Soybean	343
Roots and Tubers	81.4
Vegetables	36.86
Other plant foods	240.65
Beef	119
Mutton	193
Pig meat	213
Poultry	243
Egg	143
Milk	61
Fish and crustaceans	92.5

**Table A2:** Livestock food meat/egg/milk ratios.

Food Type	Meat/Egg/Milk Ratio
Beef	12.5
Mutton	5
Pig meat	3.2
Poultry	1.9
Egg	2
Milk	2.5
Fish and crustaceans	1.8

**Table A3:** Projected future population data for Southeast Asia *.

Region/Country	2020	2050	2100
Cambodia	1671.9	2186.1	2135.5
Indonesia	27,352.4	33,090.5	32,078.2
Laos	727.6	948	842.4
Malaysia	3236.6	4055	4007.8
Myanmar	5441	6225.3	5529.9
The Philippines	10,958.1	14,448.8	14,632.7
Thailand	6980	6594	4601.6
Timor-Leste	131.8	201.9	237.3
Viet Nam	9733.9	10,960.5	9743.7
Southeast Asia	66,862	79,400.2	74,421.5

* In tens of thousands.

**Table A4:** Scenario Analysis.

Region/ Country	Year	2020			2050			2100		
		Cultivated Land Demand for Food Consumption (×10^6^ ha)	Per Capita Cultivated Land Demand for Food (ha/per hundred)	Cultivated Land Pressure	Cultivated Land Demand for Food Consumption (×10^6^ ha)	Per Capita Cultivated Land Demand for Food (ha/per hundred)	Cultivated Land Pressure	Cultivated Land Demand for Food Consumption (×10^6^ ha)	Per Capita Cultivated Land Demand for Food (ha/per hundred)	Cultivated Land Pressure
Cambodia	Scenario 1	1.10	6.59	0.27	1.44	6.59	0.35	1.41	6.59	0.35
	Scenario 2	1.33	7.98	0.35	1.74	7.98	0.46	1.81	7.98	0.47
	Scenario 3	1.08	6.45	0.31	1.41	6.45	0.41	1.38	6.45	0.40
Indonesia	Scenario 1	16.96	6.20	0.33	20.51	6.20	0.40	19.89	6.20	0.39
	Scenario 2	18.42	6.73	0.37	22.28	6.73	0.45	21.60	6.73	0.44
	Scenario 3	10.08	3.69	0.22	12.20	3.69	0.27	11.82	3.69	0.26
Laos	Scenario 1	0.59	8.08	0.34	0.77	8.08	0.45	0.68	8.08	0.40
	Scenario 2	0.71	9.69	0.30	0.92	9.69	0.39	0.82	9.69	0.35
	Scenario 3	0.56	7.71	0.26	0.73	7.71	0.34	0.65	7.71	0.31
Malaysia	Scenario 1	3.19	9.86	0.39	4.00	9.86	0.48	3.95	9.86	0.48
	Scenario 2	2.85	8.80	0.41	3.57	8.80	0.51	3.53	8.80	0.50
	Scenario 3	1.74	5.37	0.27	2.18	5.37	0.34	2.15	5.37	0.34
Myanmar	Scenario 1	6.82	12.54	0.55	7.81	12.54	0.62	6.93	12.54	0.55
	Scenario 2	6.32	11.62	0.51	7.23	11.62	0.58	6.43	11.62	0.52
	Scenario 3	4.03	7.41	0.36	4.62	7.41	0.41	4.10	7.41	0.36
The Philippines	Scenario 1	12.37	11.29	1.13	16.31	11.29	1.49	16.51	11.29	1.51
	Scenario 2	14.98	13.67	1.42	19.75	13.67	1.88	20.01	13.67	1.90
	Scenario 3	9.86	9.00	1.03	13.00	9.00	1.36	13.17	9.00	1.38
Thailand	Scenario 1	4.55	6.51	0.21	4.29	6.51	0.20	3.00	6.51	0.14
	Scenario 2	5.46	7.83	0.29	5.16	7.83	0.27	3.60	7.83	0.19
	Scenario 3	4.10	5.87	0.24	3.87	5.87	0.23	2.70	5.87	0.16
Timor-Leste	Scenario 1	0.20	15.50	0.89	0.31	15.50	1.36	0.37	15.50	1.60
	Scenario 2	0.26	20.05	1.94	0.40	20.05	2.97	0.48	20.05	3.49
	Scenario 3	0.18	14.00	1.49	0.28	14.00	2.28	0.33	14.00	2.68
Vietnam	Scenario 1	8.38	8.60	0.71	9.43	8.60	0.80	8.38	8.60	0.71
	Scenario 2	9.59	9.85	0.70	10.79	9.85	0.79	9.60	9.85	0.70
	Scenario 3	6.16	6.33	0.50	6.94	6.33	0.56	6.17	6.33	0.50
Southeast Asia	Scenario 1	49.70	7.43	0.41	59.01	7.43	0.48	55.31	7.43	0.45
	Scenario 2	56.86	8.50	0.47	67.53	8.50	0.56	63.29	8.50	0.52
	Scenario 3	38.83	5.81	0.32	46.11	5.81	0.38	43.22	5.81	0.35
